# Two Huge Maxillofacial Osteoma Cases Evaluated By Computed Tomography

**DOI:** 10.5812/iranjradiol.4588

**Published:** 2011-12-25

**Authors:** Samira Saati, Nafiseh Nikkerdar, Amin Golshah

**Affiliations:** 1 Department of Oral and Maxillofacial Radiology, School of Dentistry, Boali University of Medical Sciences, Hamedan, Iran; 2 Department of Oral and Maxillofacial Radiology, School of Dentistry, Kermanshah University of Medical Sciences, Kermanshah, Iran; 3 Department of Orthodontics, School of Dentistry, Kermanshah University of Medical Sciences, Kermanshah, Iran

**Keywords:** Osteoma, Tomography, X-Ray Computed, Ethmoid Sinus, Mandible

## Abstract

Osteomas are benign osteogenic neoplasms or hamartomas with a very slow growth rate. Osteoma is the most common mesenchymal neoplasm of the paranasal sinuses. In the jaws, the mandible is more commonly involved than the maxilla. Osteomas may occur at any age, but most frequently are found in individuals older than 40 years. Although most osteomas are small, some may become large enough to cause severe damage, especially those that develop in the frontoethmoid region. Osteomas composed solely of compact bone are uniformly radiopaque and those containing cancellous bone show evidence of internal trabecular structure. To determine and evaluate the exact extension and internal structure of these lesions, computed tomography (CT) is a more useful imaging modality in comparison to conventional radiography. Hereby, we discuss clinical and imaging features of two osteomas (one in the ethmoid sinus and the other in the mandible) along with the main differential diagnoses and pathologic features.

## 1. Introduction

Osteoma is a slow growing, benign and rare encapsulated bone neoplasm located in the bone tissue of the skull and the face [1][2]. It may occur as solitary or multiple lesions on a single or numerous sections of the bone. The tumor may arise from cartilage or embryonal periosteum. “It may arise from the endosteal or periosteal surface” [3][4][5][6]. One of the major differences of osteoma from other bony exostoses is the ability of this lesion to continue growing during adulthood [3][5][7]. This is more common in men [1].

Histologically, osteoma may be of two types:

1) Compact or “ivory” and 2) Cancellous, trabecular or spongy [1][8][9]. The compact osteoma comprises dense bone with few marrow spaces and only few osteons. The cancellous osteoma is characterized by bony trabeculae and fibrofatty marrow enclosing osteoblasts with an architecture resembling mature bone [10][11]. “Recommended treatment is surgery, recurrence is rare and there are no reports of malignant transformation” [10][12].

Most osteomas are small; however, in rare cases they may become large enough to cause displacement and damage to adjacent structures. Although osteomas may occur at any age, they are most frequently found in people over 40 years old [1][10]. The purpose of this paper is to present the clinical, radiographic and histopathological features of two cases of huge osteoma.

## 2. Case Presentation

### 2.1. Case 1

**Figure 1:**
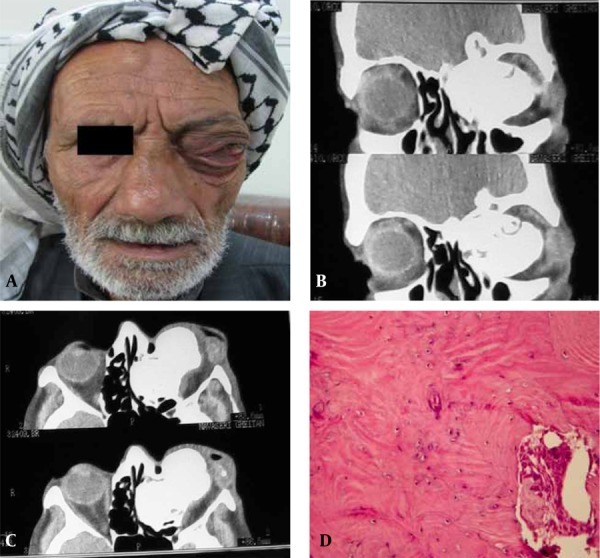
A 75-year-old man presenting with severe left globe inferolateral proptosis with lid retraction and visual loss. A, Frontal view of the patient; B, Coronal CT showing a well-defined markedly radiodense lesion; C, Axial CT shows that the mass has destroyed lamina papyracea and has extended to the orbit; D, Microscopically the lesion appears as mature bone.

A 75-year-old man was referred to the department of otolaryngology of Imam Hospital of Jundi Shapur Ahvaz University of Medical Sciences complaining of headache. On clinical examination, the left globe had severe pro-ptosis causing visual loss and lid retraction (Figure 1A). This problem had a 10-year duration. The patient had no history of previous facial trauma. Computed tomography (CT) of the orbit revealed a 5 × 3 cm well-defined markedly radiodense lesion arising from the left frontoethmoid sinus (Figure 1B). The mass had destroyed the lamina papyracea, the left rectus muscles, the left optic nerve and the left nasal wall (Figure 1C). A working diagnosis of osteoma was made based on the clinical and radiographic findings. The lesion was removed surgically under generalized anesthesia. Microscopic examination showed mature bone containing lacunae and marrow spaces (Figure 1D). A diagnosis of compact osteoma was made. The patient was examined in order to detect any osteomas in the other bones and also the polyps of the small and large intestine. The answer was negative.

### 2.2. Case 2

A 26-year-old woman came to the department of oral and maxillofacial surgery of Imam Khomeini Hospital of Jundi Shapur Ahvaz University of Medical Sciences for evaluation of a one-year-duration swelling in the left mandible which had grown slowly. She complained of dull pain in the left mandible in the last two weeks. A mild facial asymmetry was observed. Clinical examination revealed a painful firm well-circumscribed palpable mass in the buccal and lingual vestibule of the left mandible. The lesion was covered by normal mucosa. The patient had no paresthesia. The patient was in good health with a history of previous first molar extraction a few months ago.

**Figure 2:**
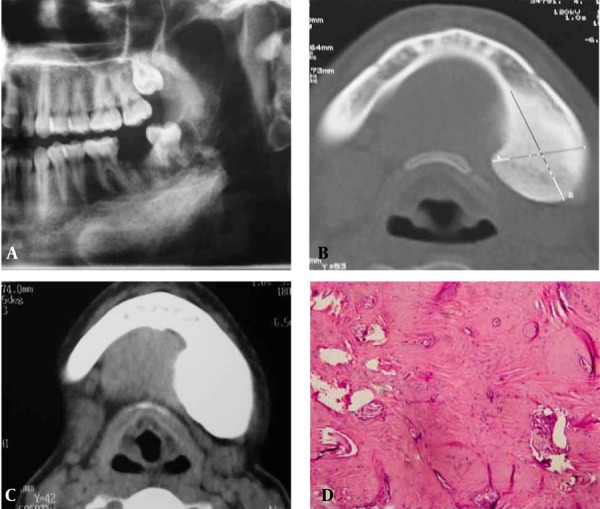
A 26-year-old woman with swelling in the left mandible A, A moderately well-defined radiopaque lesion was seen adjacent to the left inferior border of the mandible; B, Axial CT showing a well-defined radiodense lesion on the left lingual aspect; C, CT with enhancement; D, Microscopically, the marrow spaces and lacunae of the mature bone were seen.

On panoramic radiography, a moderately well-defined radiopaque lesion was seen adjacent to the inferior border of the left mandible extending from the first premolar to the third molar, not associated with the teeth and with some degree of extension to the ramus (Figure 2A). CT revealed a 3.9 × 4.2 cm well-defined, radiodense lesion arising from the left lingual aspect of the mandible displacing the lingual and buccal cortex (Figure 2B and 2C). Under local anesthesia, the incisional biopsy was performed.

Microscopic examination revealed mature bone with prominent compact components (Figure 2D). The histological features indicated the diagnosis of compact osteoma. Gardner’s or polyposis syndrome was ruled out. There was no osteoma in the other bones.

## 3. Discussion

Osteomas, which are benign, slow-growing and well-defined neoplasms may originate from membranous maxillofacial bones [1][12]. Osteoma is fairly common in the paranasal sinuses. They are more common in the frontal and ethmoid sinuses than in the maxillary sinuses [1].

The tumors are often asymptomatic and are usually detected as an incidental finding on radiographic examinations, “revealed in roughly 1% of routine scans” [13][14][15][16]. “Headache [15], epistaxis [17][18], visual changes, pain and proptosis” are the most common symptoms of unusual tumors inside the paranasal sinuses [14][19]. Osteomas are frequently accompanied by chronic inflammation of the adjacent mucous membranes lining the sinuses and by mucoceles [16].

In the frontal sinus, an osteoma can cause erosion of the posterior wall, resulting in spontaneous pneumocephalus and cerebrospinal fluid (CSF) rhinorrhea [20]. Obstruction of the draining ducts can facilitate the development of sinusitis or formation of a mucocele [20]. “Lesions larger than 3 cm in diameter are considered giant tumors” [17].

The mandible is more commonly involved than the maxilla. They usually occur in the posterior region of the mandible on the lingual side of the ramus or on the inferior mandibular border below the molars [1]. Other locations include the condylar and coronoid region. Structurally, osteomas are divided into three types: those composed of compact bone (ivory), those composed of cancellous bone and those composed of a combination of compact and cancellous bone. Osteomas may have osteoblastoma-like areas and distinguishing it from true osteoblastoma may be challenging. Some believe osteomas with osteoblastoma-like features behave more aggressively. Cortical-type osteomas develop more often in men while, women have the highest incidence of the cancellous type [1].

CT, particularly three-dimensional CT scans, is so useful in defining the exact extension of the tumor and to determine the position of the lesion in relation with adjacent anatomical structures, when removal of the lesion is considered [14][16][17][18].

The differential diagnosis includes exostoses–bony excrescences considered as hamartomas that stop growing after puberty, but osteomas may continue growth after puberty; peripheral ossifying fibroma–a reactive focal lesion; periosteal osteoblastoma; osteoid osteoma–that occur in young patients and are rare in the maxillofacial regions and parosteal osteosarcoma–that present as painful destructive masses with rapid growth [1]. The appearance and homogeneity of osteoma is not difficult to characterize and diagnose.

Osteomas involving the condylar head may be difficult to differentiate from osteochondromas, osteophytes or condylar hyperplasia and those involving the coronoid process may be similar to osteochondromas.

A person who manifests with multiple intraoral or head and neck osteomas requires further radiographic work up to rule out Gardner’s syndrome. This syndrome, consisting of multiple epidermoid or sebaceous cysts, supernumerary teeth, retinal pigmentation and intestinal polyposis, necessitates a gastrointestinal radiographic evaluation because the polyps involved are premalignant [20]. The treatment for osteoma is surgical excision, particularly if there is painful or active lesion growth [21].

CT scans, particularly CT scan in bone window and magnetic resonance imaging (MRI) give very good diagnostic possibilities, but plain radiography is also sufficient for the purpose of post operative follow-up. “Scan should be performed at least in six-month intervals during the first few years after surgery” [22]. Recurrence after surgical procedure is rare.
